# Manifestations bucco-dentaires de la sclérodermie systémique

**DOI:** 10.11604/pamj.2013.16.114.3065

**Published:** 2013-11-24

**Authors:** Bouomrani Salem, Bel Hadj Ali Rim, Ben Khoud Sihem, Béji Maher

**Affiliations:** 1 Service de Médecine Interne, hôpital Militaire de Gabes 6000, Tunisie; 2 Service de Chirurgie Dentaire, hôpital Militaire de Gabes 6000, Tunisie

**Keywords:** Sclérodermie systémique, microstomie, parodontite, systemic sclerosis, microstomia, periodontitis

## Abstract

Nous rapportons l'observation d'une jeune femme de 26 ans ayant une sclérodermie systémique diffuse présentant une atteinte bucco-dentaire complexe: microstomie, hyperplasie gingivale, parodontite, dépôts tartriques, caries multiples et chevauchement dentaire antéro-inférieur. Nous discuterons à travers cette observation les manifestations bucco-dentaires de cette connectivite qui sont loin d’être rares mais souvent négligées par les cliniciens malgré leur retentissement fonctionnel majeur. Il convient de surveiller régulièrement l’état bucco-dentaire chez tout patient sclérodermique afin de diagnostiquer précocement ces atteintes. Diagnostiquées à un stade tardif les complications bucco-dentaires de la sclérodermie seront très difficiles à traiter.

## Introduction

La sclérodermie systémique (SS) ou encore sclérose systémique est une connectivite systémique caractérisée par une atteinte cutanée prédominante et spécifique associée à des manifestations viscérales pouvant être mortelles [1–3].

Le substratum anatomique commun à ces atteintes est une fibrose et sclérose du tissu conjonctif avec une hyperproduction de collagène à laquelle s'associent une vasculopathie assez spécifique de la microcirculation (micro-angiopathie sclérodermique) et un infiltrat inflammatoire [1–3]. Son étiopathogénie est toujours mal élucidée; elle serait multifactorielle faisant intervenir des facteurs environnementaux et dysimmunitaires sur un terrain génétiquement prédisposé [4].

Son diagnostic est principalement clinique et ne pose pas de problèmes en dehors des formes exceptionnelles sans atteinte cutanée dites « sine scleroderma ». Selon l'extension de la sclérose cutanée on distingue deux grands types de SS: les SS limitées où l'atteinte cutanée se limite aux extrémités de membres et les SS diffuses plus graves avec une atteinte tronculaire et proximale des racines de membres [1–3, 5].

Les manifestations bucco-dentaires sont rarement étudiées et souvent négligées par les cliniciens malgré leur retentissement fonctionnel très marqué et l'handicap fonctionnel majeur qu'elles entrainent [6]. Nous étudierons, à travers cette observation, l'ensemble des atteintes bucco-dentaires au cours de cette connectivite ainsi que leurs particularités thérapeutiques et pronostiques.

## Patient et observation

Patiente âgée de 26 ans, présentant une sclérodermie systémique diffuse évoluant depuis 2006 et diagnostiquée devant une sclérose cutanée proximale et tronculaire associée à des télangiectasies diffuses, une hypomotilité ‘sophagienne, une calcinose sous cutanée diffuse, des ulcérations pulpaires et des auto-anticorps anti nucléaires et anti Scl 70 positifs, fût adressée pour une mise en état de la cavité buccale. La patiente était traitée au long cours par colchicine^®^ (1 mg/j), Inexium^®^ (20 mg/j), Nifédipine^®^ (30 mg/j) et Cortancyl^®^ à faible dose (10 mg/j).

**Figure 1:**
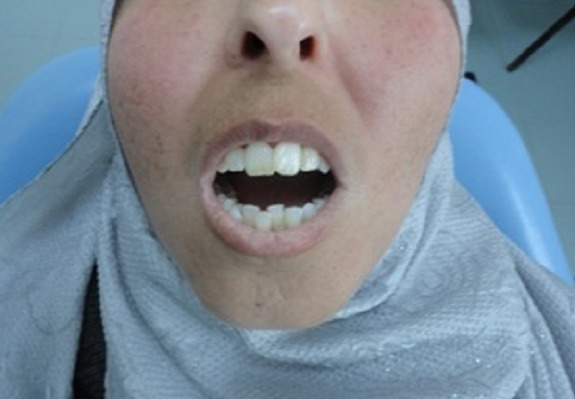
Microstomie, limitation de l'ouverture buccale à deux travers de doigt

**Figure 2:**
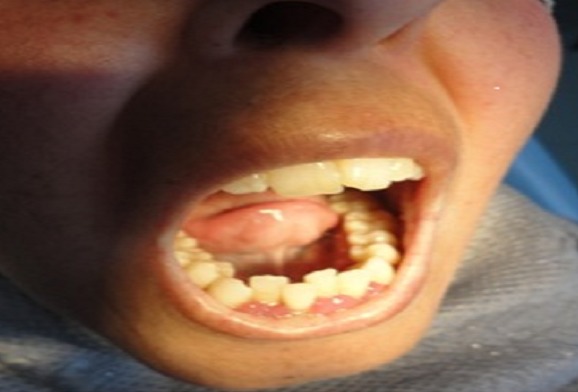
Frein lingual épaissi rendant la protraction linguale difficile

**Figure 3:**
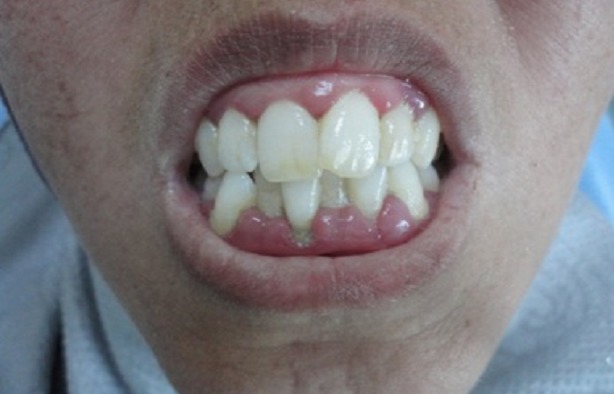
Chevauchement dentaire antéro-inférieur avec une hyperplasie gingivale

**Figure 4:**
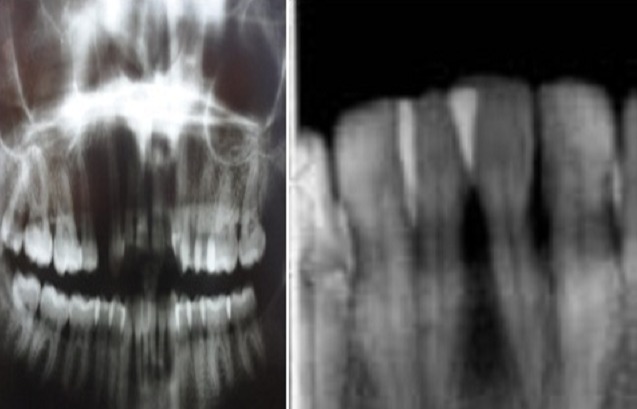
Les radiographies-X standards (incidences panoramique et retro dentaire): signes de parodontite

L'inspection du visage révélait un faciès caractéristique figé et momifié: peau pâle, rides d'expression effacées, paupières rétractées, nez effilé avec des narines pincées et lèvres pâles, fines, soulignées par des ridules radiaires. L'examen des articulations temporo-mandibulaires (ATM) était sans anomalies. La patiente décrivait par ailleurs une gêne à la mastication avec un syndrome algique myofacial en rapport avec la sclérose de la peau du visage et du cou, des muscles et des ligaments. L'ouverture buccale était limitée (microstomie à deux travers de doigt) (Figure 1). L'hygiène buccale était moyenne (entravée par la microstomie et la dextérité manuelle réduite). La muqueuse buccale était pâle, sèche avec présence de multiples télangiectasies jugales. La langue était sèche, dépapillée, de volume et mobilité réduits et le frein lingual épaissi rendant la protraction linguale difficile (Figure 2). L'examen dentaire montrait un chevauchement dentaire antéro-inférieur, des lésions cervicales non carieuses au niveau des prémolaires supérieures et inférieures et des caries délabrantes de la 14,28 et 38 (Figure 3). L'examen gingival trouvait une gencive molle, lisse et irrégulière avec des hypertrophies papillaires prononcées au niveau des blocs incisivo-canins associées à des dépôts tartriques sus et sous gingivaux. La pression digitale laissait apparaitre un exsudat purulent sur le rebord gingival. Le sondage déterminait l'existence de poches parodontales supra-osseuses et de fausses poches; Il était douloureux, occasionnant une gingivorragie importante. Les radiographies-X standards (incidences panoramique et retro-alvéolaire) révélaient une faible densité osseuse en regard des canaux mandibulaires, des élargissements ligamentaires surtout au niveau des dents mandibulaires et une lyse osseuse horizontale au niveau du secteur antéro-inférieur posant le diagnostic d'une « parodontite manifestation de maladie systémique » selon la classification d'Armitage GC 1999 (Figure 4).

La prise en charge thérapeutique consistait en un détartrage surfaçage radiculaire, une restauration des mylolyses, des extractions des racines de la 14, 28 et 38 sous antibioprophylaxie (Amoxicilline^®^ 2 g, une heure avant l'intervention), un curetage parodontal et des gingivectomies. Un traitement prothétique fût envisagé pour le remplacement de la 14. Un complément physique fût aussi conseillé: motivation à l'hygiène bucco-dentaire, enseignement d'une bonne méthode de brossage et apprentissage d'exercices d'augmentation de l'ouverture buccale.

## Discussion

Rapportées pour la première fois en 1968 [7], les complications dentaire de la SS restent jusqu’à nos jours mal connues et peu étudiées malgré leurs intérêts considérables aussi bien diagnostique que pronostique [6] et leur fréquence non négligeable [8]: 100% des patients ayant une sclérodermie juvénile de Trainito S. ont signalé au moins une complication odonto-stomatologique; les anomalies dentaires proprement dites ont été objectivé chez 63% d'entre eux [6].

En effet l'intérêt diagnostique et pronostique des atteintes bucco-dentaires au cours de la SS fût déjà connu depuis longtemps et même assimilé, par certains auteurs, à celui de l'atteinte eosophagienne au cours de cette connectivite [9]. Ces atteintes peuvent survenir aussi bien dans les formes diffuses de SS [6, 10] que celles limitées; en particulier le CREST syndrome [6, 11–13]. Elles peuvent se voir aussi bien chez l'adulte que chez l'enfant et l'adolescent [6, 14].

Au cours de la SS, l'atteinte buccale la plus caractéristique associe une microstomie, une microcheilie et des plis radiaires péri buccaux rentrant dans le cadre du facies sclérodermique typique [15]. A coté de ce facies caractéristique, on peut citer les télangiectasies buccales [10], l'hyposialie et/ou la xérostomie rentrant dans le carde d'un syndrome sec secondaire [14], les dysfonctions temporo-mandibulaires [6, 16] avec au stade ultime une arthrite destructrice et rapidement progressive des articulations temporo-mandibulaires [6], les condylites mandibulaires bilatérales [17] et le cancer de la langue dont la fréquence se trouve significativement augmentée chez les sclérodermiques avec une ouverture buccale inférieure à 30 mm [18].

Les atteintes dentaires proprement dites sont aussi rapportées au cours de la SS [6]. Parmi ces atteintes on retrouve l'instabilité dentaire [12, 14], les caries dentaires [19], les anomalies de l’éruption dentaire [6] et une fréquence anormalement augmentée de dents cariées, obturées et absentes mieux connue sous le nom de « Increased Decayed, Missing, and Filled Teeth: DMFT » [14].

Les parodontites sont aussi relativement fréquentes au cours de la SS [14]. Elles sont classiquement de type IV: parodontites-manifestations d'une maladie systémique, selon la dernière classification de “l'International Workshop for Classification of Periodontal diseases and condition” de 1999 [20]. De même que l’élargissement des ligaments péri-dontaux [14] et la réduction significative de la distance inter incisives [10, 14].

Les lésions dentaires au cours de cette connectivite sont favorisées par plusieurs facteurs; en particulier l'hyposialie et/ou la xérostomie [14, 21], les résorptions osseuses mandibulaires [14, 21], la fibrose gingivale [21] et les infections stomatologiques surtout mycosiques [22]. L'ensemble de ces remaniements cutanéo-muqueux, osseux et gingivaux rend compte des difficultés rencontrées par les cliniciens lors des interventions bucco-dentaires chez les sclérodermiques [21, 23].

Actuellement les recommandations de la prise en charge spécifique des complications bucco-dentaires de la SS sont bien codifiées (Table 1). Elles étaient développées initialement à la suggestion d'un groupe français d'experts multidisciplinaires puis ultérieurement validées par un comité de lecteurs et par “l'American College of Rheumatology: ACR” [18]. Le traitement reste avant tout symptomatique comme celui du syndrome sec primitif en insistant sur l'hygiène bucco-dentaire et le suivie stomatologique régulier [8, 12, 18]. Les lèvres et les commissures doivent être bien lubrifiées par la vaseline.

**Tableau 1:** Niveau de recommandations pour la prise en charge des complications bucco-dentaires au cours de la sclérodermie systémique

Type de l'atteinte bucco-dentaire	Prise en charge	Niveau de recommandations (Evidence base level)
**Syndrome sec**	Teinture de pilocarpine, pilocarpine, anétholtrithione, salive artificielle en spray	Recommandation d'experts de routine
**Parodontites**	Education à l'hygiène, antibiothérapie, détartrage et procédures d'aménagement des racines et entretien semestriel	Niveau 1
**Plaques dentaires et/ou hémorragies gingivales sur hyperplasie ou induite par les AVK**	Education à l'hygiène, détartrage et procédures d'aménagement des racines et bains de bouche à l'acide tranexamique	Niveau 1
**Caries**	Soins conservateurs et prophylaxie dentaire avec les fluorures	Recommandations d'experts de routine
**Résorption osseuse mandibulaire**	Pas de traitement - Surveillance simple	Recommandations d'experts de routine
**Microstomie sévère (<30mm)**	Rééducation avec des exercices d’élongation pendant 3 mois au moins	Niveau 2
**Edentation**	Prothèses dentaires amovibles, implants dentaires (en fonction des comorbidités, traitement par bisphosphonates et le niveau d'hygiène)	Recommandations d'experts de routine
**Rides péri buccaux**	Laser CO_2_ pulsé	Niveau 4

Une radiographie panoramique systématique est recommandée chez tout patient sclérodermique pour le diagnostic précoce des caries et des anomalies ostéo-articulaires [18].

Les soins dentaires classiques peuvent être pratiqués sans risque chez les sclérodermiques, y compris l'utilisation de matériaux de résine, d'amalgames, verres ionomères ou de couronnes dentaires. Ces soins doivent être effectués quadrant par quadrant afin d’éviter de longues séances souvent mal tolérées vue la limitation de l'ouverture buccale [18].

Une couverture antibiotique s'avère indispensable pour ces soins surtout en présence d'infection buccale et/ou dentaire évolutive; en particulier dans les parodontites agressives généralisées [24]. L'association amoxicilline-métronidazole représente le traitement de première ligne. Les macrolides sont une alternative efficace chez les sclérodermiques allergiques ou traités par méthotrexate [18].

Les prothèses partielles amovibles ainsi que la kinésithérapie de réhabilitation buccale sont fortement recommandées; l'indication d'implants dentaires dépend principalement de la sévérité de la SS, des comorbidités et de l’éventualité ou non d'un traitement par bisphosphonates [18]; vue le risque potentiel d'ostéonécrose mandibulaire avec ces molécules. Il faut toute fois signaler que la présence d'une atteinte gingivale marquée avec fibrose et hypoperfusion contre indique toute chirurgie muco-gingivale chez le sclérodermique du faite de son efficacité très limitée et du risque accru de complications post opératoires [18].

## Conclusion

Les complications bucco-dentaires sont loin d’être rares au cours de la SS si recherchées systématiquement. Elles se caractérisent par un retentissement fonctionnel et esthétique très marqué. Ces atteintes nécessitent un dépistage systématique, des contrôles réguliers cliniques et radiographiques et une prise en charge multidisciplinaire précoce et adaptée.
